# Potential of pest regulation by insectivorous birds in Mediterranean woody crops

**DOI:** 10.1371/journal.pone.0180702

**Published:** 2017-09-06

**Authors:** José M. Rey Benayas, Jorge Meltzer, Daniel de las Heras-Bravo, Luis Cayuela

**Affiliations:** 1 Life Sciences Department-Ecology Unit, Alcala University, Alcalá de Henares, Spain; 2 International Foundation for Ecosystem Restoration, Madrid, Spain; 3 Department of Biology, Geology, Physics and Inorganic Chemistry, Rey Juan Carlos University, Móstoles, Spain; University of South Carolina, UNITED STATES

## Abstract

Regulation of agricultural pests managing their natural enemies represents an alternative to chemical pesticides. We assessed the potential of insectivorous birds as pest regulators in woody crops located in central Spain. A total of 417 nest boxes installed in five field study sites (one vineyard, two fruit orchards, and two olive groves) were monitored for use and breeding of insectivorous birds and other species for four consecutive years (2013–2016). At all field sites except the two olive groves, where birds never occupied the nest boxes, predation experiments were conducted with Greater wax moth (*Galleria mellonella*) sentinel caterpillars, and food consumption by birds was estimated. Nesting of insectivorous birds, chiefly Great tit (*Parus major*), and sparrows (*Passer domesticus* and *P*. *montanus*) increased over time, averaging 60% per field site in the vineyard and fruit orchards by the fourth year. Use of nest boxes by sparrows and by Garden dormouse (*Eliomys quercinus*) was high at the fruit orchards (70%) and the vineyard (30%), respectively. Micro-habitat characteristics (nest box level) and meso-habitat characteristics (patch level) strongly affected use of nest boxes and bird breeding (i.e. number of laid eggs and produced chicks) in different years. Distance to natural or semi-natural vegetation did not consistently affect bird breeding, nor did we see consistent evidence of competition between adjacent breeding birds. Predation rates of sentinel caterpillars were approximately one-third higher near boxes with nesting birds (31.51 ± 43.13%) than at paired distant areas without nest boxes (22.45% ± 38.58%). Food consumption by insectivorous birds per ha and breeding season were conservatively estimated to range from 0.02 kg in one fruit orchard to 0.15 kg in the vineyard. We conclude that installation of nest boxes in Mediterranean woody crops enhances populations of insectivorous birds that regulate pests, but that the effects are moderate and highly context-dependent.

## Introduction

The need to maintain and enhance crop yield has led to an increase in agrochemicals use throughout the approximately 12% of the Earth’s ice-free land that is cultivated [1]. In particular, more than 2.4 billion tons of pesticides were used worldwide in 2007 [2]. Despite their advantages, pesticides can harm biodiversity [3, 4], including organisms beneficial to agricultural production itself (natural pest enemies, pollinators and decomposers [5]), and they can degrade the overall environment by polluting soil and water [6] and promoting soil erosion [7]. Pesticides can also threaten human health [8], and they can become ineffective when pests develop resistance [9]. An alternative to pesticide use is to regulate pests by maintaining or enhancing their natural enemies [10]. This strategy offers a more sustainable agricultural management because it can simultaneously promote biodiversity and ecosystem services other than crop production [11, 12, 13].

One third of the commercially available pesticides target arthropods [2], leading to the loss of an average of 15% of crops worldwide [14]. Crop insect pests have many natural enemies that could be exploited to manage the pest population as an alternative to pesticides; these enemies comprise several taxa, including bacteria, fungi, invertebrates, and vertebrates, which engage in diverse trophic relationships with pests, including predation, parasitism, parasitoidism, and disease. Previous research of pest enemies has focused mostly on microorganisms and invertebrates [15, 16, 17], while less attention has been paid to vertebrate species (e.g. birds) for crop pest regulation, particularly in temperate areas (but see [18, 19]).

This research gap is important to address given that several studies have already suggested that insectivorous birds can be effective for controlling crop pests. For example, Great tits (*Parus major*) significantly reduced caterpillar damage by 19% and increased fruit yield by 66% in German commercial apple orchards [20]. The presence of nest boxes occupied by insectivorous birds in a Californian vineyard increased the average removal rate of Beet armyworms (*Spodoptera exigua*) by a factor of 3.5 [21]. In Costa Rica, birds reduced by half an infestation of Coffee berry borer beetles (*Hypothenemus hampei*) [22]. Excluding bats and birds from an ecosystem in Indonesia led to an increase in insect herbivore abundance and concomitant 31% decrease in final crop yield [23].

In the present study, we assessed the potential of insectivorous birds to regulate pests in Mediterranean woody crops. Woody crops cover nearly five million ha in Spain, accounting for 29.1% of cropland in the country [24]. They are the dominant vegetation in many agricultural landscapes in this and other regions of the world, and they are important habitats for species targeted by conservation efforts [25]. Woody crops occur in areas protected by the European Union Natura 2000 Network of natural protected areas [26]. They often demonstrate well sustainable agricultural practices that balance the requirement for agricultural production against the desire for wildlife conservation [27].

Here we studied vineyards (*Vitis vinifera*); orchards of Peach trees (*Prunus persica*), Prune trees (*Prunus domestica*), and Pear trees (*Pyrus communis*); and groves of Olive trees (*Olea europaea*). We installed nest boxes and conducted sentinel predation experiments for four consecutive years (2013–2016). Our specific objectives were to (1) evaluate nest box use by insectivorous birds and other species, (2) evaluate the factors that may explain breeding of insectivorous birds at nest boxes, (3) measure predation near nest boxes containing nesting birds and compare such predation with that in distant areas without nest boxes, and (4) estimate food consumption by insectivorous birds on selected farms. We hypothesized that (H1) birds’ use of nest boxes would increase with time, (H2) birds would favor habitat patches most suitable for breeding, (H3) more breeding would occur near patches of non-crop natural or semi-natural vegetation, and (H4) predation of sentinel caterpillars would be greater near nest boxes with nesting birds than in distant areas without nest boxes.

As far as we know, this is the first systematic experimental work on pest regulation by insectivorous birds in various types of woody crops within the same study. The lessons learned here may be useful to farmers and conservation practitioners trying to move towards more wildlife-friendly farming.

## Material & methods

### Study sites

**Fig 1:**
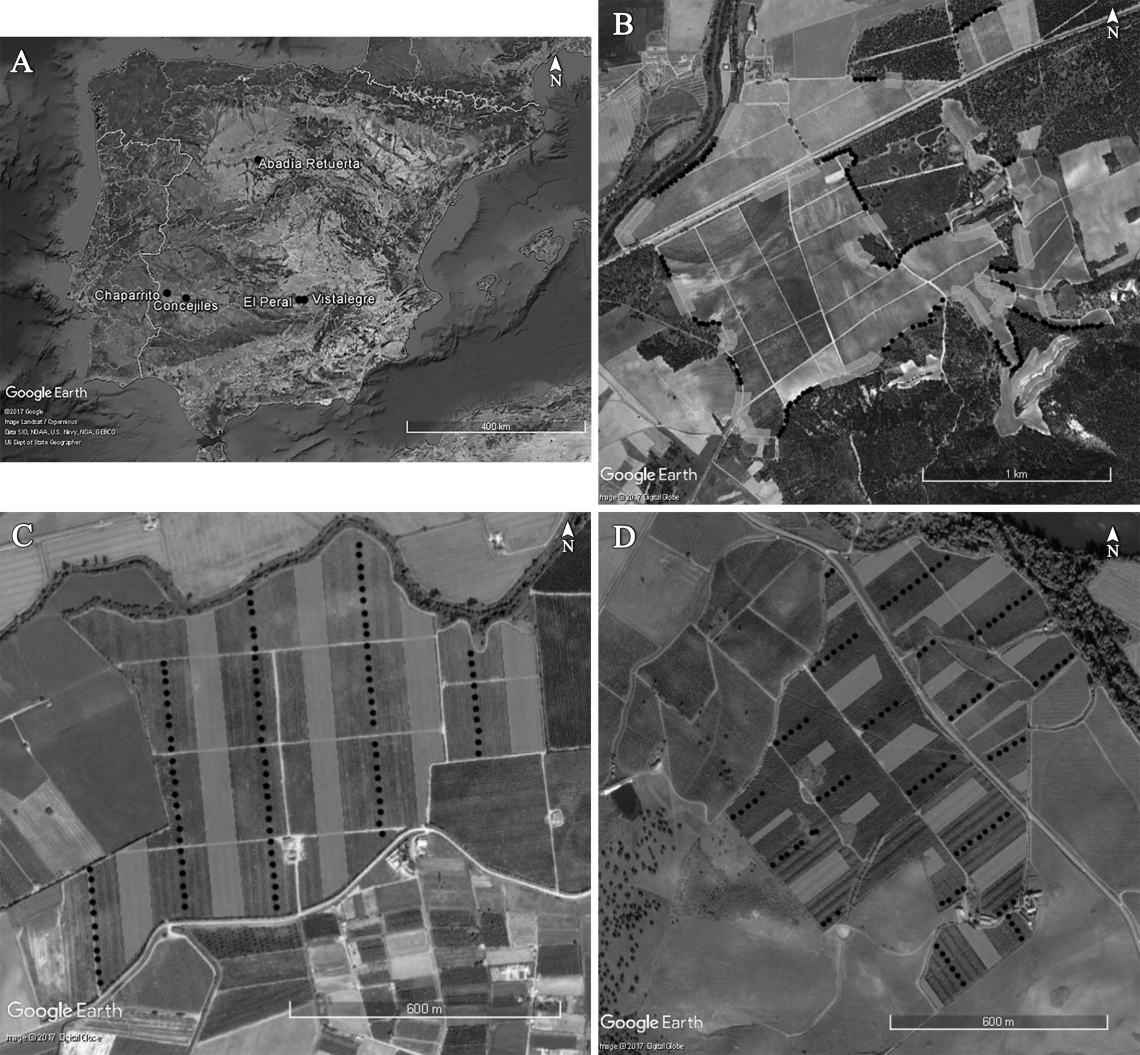
(A) Location of the five study sites in central Spain, namely the Abadía Retuerta vineyard, the Concejiles and Chaparrito fruit orchards and the Vistalegre and El Peral olive groves. (B) Distribution of the initial 164 nest boxes (black dots) in the Abadía Retuerta vineyard. (C) Distribution of the initial 101 nest boxes (black dots) in the Concejiles fruit orchard. (D) Distribution of the initial 111 nest boxes (black dots) in the Chaparrito fruit orchard. Light gray polygons indicate distant areas without nest boxes used for sentinel caterpillar experiments. Images were taken from [28] under a CC BY license, with permission from Google Maps/Google Earth (URL: https://www.google.es/intl/es/permissions/geoguidelines.html), original copyright 2015. The four panels are not identical to the original images, and are therefore for illustrative purposes only.

We studied the following five sites in Central Spain (Fig 1A): the 203-ha Abadía Retuerta vineyard (41.61° N, -4.41° W); the 62-ha Concejiles fruit orchard with Peach and Prune trees (38.83° N, -6.28° W); the 80-ha Chaparrito fruit orchard with Peach, Prune and Pear trees (38.94° N, -6.76° W); a 4.6-ha olive grove (38.81° N, -3.23° W); and a second olive grove of 1.9 ha (38.81° N, -3.36° W). No occupancy of insectivorous birds was detected at either olive tree grove throughout the four-year study, probably because they lay >2 km from suitable bird habitat and because the nest boxes were not covered by vegetation [15, 29–31]. Therefore these two sites were not considered further in this study.

The vineyard was surrounded by Stone pine *Pinus pinea* plantations of different age (the border between the vineyard and pine plantations was 5,200-m long). The vineyard was also surrounded by natural Mediterranean vegetation dominated by Holm oak *Quercus ilex* subsp. *ballota* (the border was 3,200-m long), and there was a 1,350-m strip of riparian forest on the northwestern side (Fig 1B). The Concejiles fruit orchard was surrounded by rainfed cropland with sparse Holm oak *Quercus ilex subsp*. *rotundifolia* at a distance of 150 m on all sides except the northeastern side with presence of a 700-m strip of riparian vegetation and planted eucalyptus. *Retama sphaerocarpa* shrubland and Holm oak woodland lay, respectively, 380 and 700 m from the southwestern edge of this site (Fig 1C). The Chaparrito fruit orchard was surrounded by other fruit orchards and open rice fields; the northern edge of this site shared a 1,450-m border with a strip of degraded riparian vegetation (Fig 1D). Climate at all study sites is Mediterranean, with warm and dry summers and cold winters (S1 Table).

### Setting and monitoring of nest boxes

Since the crops at the study sites lacked appropriate cavities to serve as nesting sites for insectivorous birds, we installed 376 nest boxes in total to favor their breeding [32] at the three sites between January and mid-February in 2013. Nest boxes in the vineyard were made from wood; those in the fruit orchards were made from wood or pressed cardboard. The hole in the nest boxes had a diameter of 3.5 cm, widely used to favor nesting of the most common insectivorous bird species at the study sites, namely the Great tit (*Parus major)* and Blue tit (*Cyanistes caeruleus)*. During the study, we noticed that other bird species and rodents, particularly the Garden dormouse (*Eliomys quercinus)*, also used the installed nest boxes (see below). Thus, in February 2016 (the last year of the study), we left the nest boxes uncovered before the breeding season in order to reduce occupancy by Garden dormouse.

All study sites included a treatment area with nest boxes as well as a distant area without nest boxes, and the two areas were separated by a buffer area (specific distances are reported below). Distant areas without nest boxes were used only for predation experiments (see below). In all treatment areas, the high number of nest boxes assured that availability of sites was not a limiting factor for bird breeding. Nevertheless, the separation between adjacent nest boxes was consistently 25 m because breeding individuals of small insectivorous birds regularly patrol small territories around their nest [33, 34]. In other respects, the layout and characteristics of the “treatment area-buffer area-distant area” differed across study sites, reflecting differences in crop type, area, configuration, and surrounding vegetation.

As a preliminary trial, we installed 63 nest boxes at the maximum vine height of 1.5 m inside the vineyard in february 2012. None of the boxes was occupied by birds, and 19 were used by wasps for nesting. Vine leaves sprout in late spring, so nest boxes were not covered when birds were exploring sites for nesting. Since the vineyard interior turned out to be a highly unsuitable habitat for insectivorous birds, we installed the nest boxes in clusters at the border between the pine plantations or Holm oak forest and the individual vineyard lots; the nest boxes were oriented with the entrance holes facing the vineyard. Within a cluster, nest boxes were placed in tree branches at heights >1.5 m above the ground. In each vineyard lot, the length of the cluster with nest boxes was equal to the length of the distant area without boxes; a 50-m buffer area lay between the cluster with nest boxes and the distant area without boxes. We initially placed 164 nest boxes at this study site (Fig 1B); a few were lost each year as follows: 3 in 2013, 9 in 2014, 12 in 2015, and 14 in 2016.

The fruit orchards included individual fields planted with different tree species, and each field was sufficiently large to include at least one tree line with nest boxes and one distant tree line without nest boxes, with a buffer area at least 50-m wide separating the two tree lines. Nest boxes were placed in tree branches as high as possible, 1–2 m above the ground. At the Concejiles site, 101 nest boxes were installed initially (Fig 1C), and some were lost each year as follows: 3 in 2013, 9 in 2014, 18 in 2015, and 19 in 2016. At the Chaparrito site, 111 nest boxes were installed initially (Fig 1D), and some were lost each year: 3 in 2013, 11 in 2014, 16 in 2015, and 29 in 2016. We used a GPS to assess the exact position of every nest box at the two fruit orchards and the vineyard.

Nest boxes were monitored for four consecutive years (2013–2016) from the start of the breeding season until the end of the summer in order to capture late recruitment (objectives 1–2). Each nest box was monitored four to five times per year except in 2014 and 2016, when the breeding season was quite short. In these years, nest boxes were monitored twice in the vineyard and three times in the fruit orchards. We judged this frequency of nest visits as adequate for assessing overall use of nest boxes and their potential for bird breeding, since we were interested in analyzing the use of nest boxes and not the phenology of breeding. We distinguished the following categories of nest-box use: 0 –empty nest box, 1 –initiated but unfinished bird nest, 2 –finished bird nest, 3 –laid eggs, 4 –hatched eggs, 5 –recruited fledglings, and 6 –presence of species other than birds. The number of recruited fledglings was determined on the basis of the number of individuals that were counted in the nest box at the last visit at the end of the breeding season, thus including second lays. In the autumn after the breeding season, nest boxes were cleaned and repaired or replaced if necessary.

### Predation experiments

Over the whole study period, we conducted sentinel predation experiments using larvae of Greater wax moth (*Galleria mellonella*). These experiments were designed to test whether predation rates differed between areas near nest boxes with breeding birds (“active” nest boxes *sensu* [21]) and distant areas without nest boxes (objective 3). We performed sentinel experiments on the day immediately after the nest boxes had been monitored at each site, except at the end-of-summer monitoring. We placed two samples of 10 caterpillars each, one closer and one farther away (specific distances are provided below) from each nest box containing laid eggs or chicks. Distance between adjacent caterpillars within a sample was 8 cm. In the vineyard, the sample closer to the active nest box lay <6 m from the box, at the border between the forest or tree plantation and the vineyard; the sample farther from the active nest box lay on the nearest vine at least 15 m away from the box. In the fruit orchards, the sample closer to the active nest box was placed on the fruit tree nearest to the one holding the box, approximately 3 m away; the sample farther from the active nest box lay on a fruit tree approximately 10 m from the box. Two other samples of 10 caterpillars each were placed in the distant area without nest boxes such that the distance between samples was similar to that in the treatment area with active nest boxes. To determine predation rate, we fixed live caterpillars to twigs using pins with colored heads during the first 3 hours after sunrise. After 6–8 h of exposure, the caterpillars remaining in the sample were counted.

### Estimation of food consumption by insectivorous birds

Over the whole study period, we estimated the amount of food consumption by insectivorous birds at each field site during the breeding season (objective 4). The food consumption of each breeding pair of birds and its brood was estimated for the period from mid-March to mid-July based on published food consumption levels during the breeding season for Great tit. These levels were adjusted based on the weight of the observed breeding bird species relative to the weight of Great tit and based on the number of breeding pairs and their respective chicks. This estimated consumption was then summed for all insectivorous birds at the same field site and during the same breeding season in order to obtain the estimated total amount of food consumption.

Food consumption was estimated as follows. Great tits consume 1 Kcal per gram of weight per day [35]; in a trial of mixed insects, 1 g of dry matter food was equivalent to 5.82 Kcal, indicating that the average Great tit needs 0.17 g x 18.5 g (bird weight [36]) = 3.145 g of dry food matter per day. That author calculated a similar amount of dry food matter per gram of weight per day for Coal tit (*Periparus ater)*. Similarly, Royana (1966) [37] calculated that one Great tit chick receives on average a total of ca. 650 mg (dry weight) of food per day for its whole period in the nest, a figure consistent with a previous study [38]. Based on these previous estimates, we estimated food consumption of a breeding pair of Great tits and its chicks as follows: (3.145 g per adult and day *x* two adults *x* 120 days = 754.8 g) + (0.65 g per chick and day *x* number of chicks *x* 19 days at nest box) + (3.145 g per adult and day *x* number of recruited juveniles *x* 30 days). This 30-day figure refers to the time that fledglings and juveniles remain around their nests until the end of the breeding season. To this estimate we added the estimated consumption by breeding pairs with laid eggs but unverified hatching (754.8 g per pair and breeding season using the above calculations). The weight of other insectivorous bird species that bred in our nest boxes is smaller: 10.5 g for Blue tit, 9 g for Coal tit, and 11 g for Crested tit (*Lophophanes cristatus*) [36]. Thus, we corrected the estimated food consumption by these insectivorous bird species by determining the consumption based on Great tit weight and then multiplying by 0.57, 0.47 or 0.59, respectively. We did not estimate food consumption by sparrows (see below) since these are mostly granivorous birds with much lower potential for pest regulation than insectivorous birds. We estimated food consumption at each site on a per site basis and per ha basis.

### Statistical analyses

To address H1 –higher use of nest boxes by birds with time-, we tested whether the category of nest box use correlated between two consecutive years (Spearman’s rank correlation). For this analysis, nest boxes used by non-avian species (category 6) were considered as empty (category 0).

To test H2 and H3 –factors affecting bird breeding-, we used generalized linear mixed models (GLMMs) with a negative binomial error distribution (to account for over-dispersion in model residuals) and a log-link function. Models were fit independently for each study site; the study site was not added as a fixed factor in the models because the differences in nest-box lay impeded so. We analyzed the effects of the following three fixed factors: year, vicinity index related to occupancy of close nest boxes by birds, and distance to the closest patch or remnant of natural or semi-natural vegetation on breeding. Breeding was measured as the number of laid eggs and chicks or fledglings, including second lays (categories 3–5 of nest box use). We accounted for the random effect of nest boxes, as they were located at the same points during the study and thus offered the same microhabitat during the breeding season, potentially giving rise to data autocorrelation. An appreciable random effect in the selected models would indicate the presence of micro-habitat selection. We used year as a categorical factor. Occupancy of nest boxes in the vicinity of each target nest box was measured as the number of adjacent nest boxes occupied by birds (i.e. categories 2–5), ranging between 0 and 2. A relevant negative or positive effect of this vicinity index in the selected models would suggest, respectively, competition or selection at the meso-habitat level. Distance to natural or semi-natural vegetation was measured as a continuous variable using a geographical information system; this variable was not included in models for the vineyard, as nest boxes were placed at the border between such vegetation and vineyards.

The Akaike Information Criterion corrected for small samples (AIC_c_) was used to select among alternative models. Models that differ in AIC_c_ by more than 2 indicate that the worse model has virtually no support and can be omitted, while models that differ in AIC_c_ by less than 2 make up a confidence set of models [39]. We followed a two-step procedure for model selection [40]. First, we fitted the beyond-optimum model for fixed effects (i.e. the most complex model for fixed effects, including all their pairwise interactions) and compared models that differed in their random structure, namely: (1) no random factor; (2) the effect of nest box on the intercept; and (3) the effect of nest box on the intercept and its interaction with the vicinity index of occupancy, implying that the effect of bird occupancy of adjacent nests on bird breeding might vary randomly among nest boxes. Second, once the optimal random structure was selected, we compared an array of models that differed in their fixed-effect structure, including different combinations of the variables year, vicinity index of occupancy, distance to natural or semi-natural vegetation, and their pairwise interactions. Akaike weights (w_i_) were calculated for the confidence set of models (ΔAIC_c_ < 2) to determine the weight of evidence in favor of each model and to estimate the relative importance of each individual parameter in the set of candidate models. If no single model is clearly superior to the others in a set of models (model with w_i_ < 0.9), a (weighted) model averaging approach should be used [39]. Hence, we used the entire set of plausible models (ΔAIC_c_ < 2) to calculate model-averaged estimates for variables included in the confidence set of models and their unconditional standard errors. Residual plots were explored to assess model assumptions. Pseudo-R^2^ cannot be estimated in negative binomial GLMMs.

Finally, to test H4 –predation of sentinel caterpillars-, we used GLMM with a binomial error distribution and a logit-link function. Again, analyses were run independently for each field site. Fixed-effect predictors were treatment (i.e. active nest box vs. distant area without nest boxes) and proximity to nest box (closer vs. farther sentinel samples), whereas each nest box was used as a random factor to account for pairing of samples and potential temporal autocorrelation (i.e. observations that might have similar values across years due to the individual effect of nest boxes). The procedure implemented for model comparison and selection based on AIC_c_ was similar to that followed for testing H2 and H3 (see above). In addition, the conditional R^2^ (fixed and random effects) and marginal pseudo-R^2^ (only fixed effects) were estimated [41] for the best model at each study site.

ArcGis 10.0 software [42] was used to measure distance of nest boxes to natural or semi-natural vegetation as well as to produce maps. Statistica 7.0 [43] was used for correlation analysis, and the 'glmmADMB' [44] and 'lme4' [45] packages in R were used for GLMMs.

## Results

### Use of nest boxes

**Fig 2:**
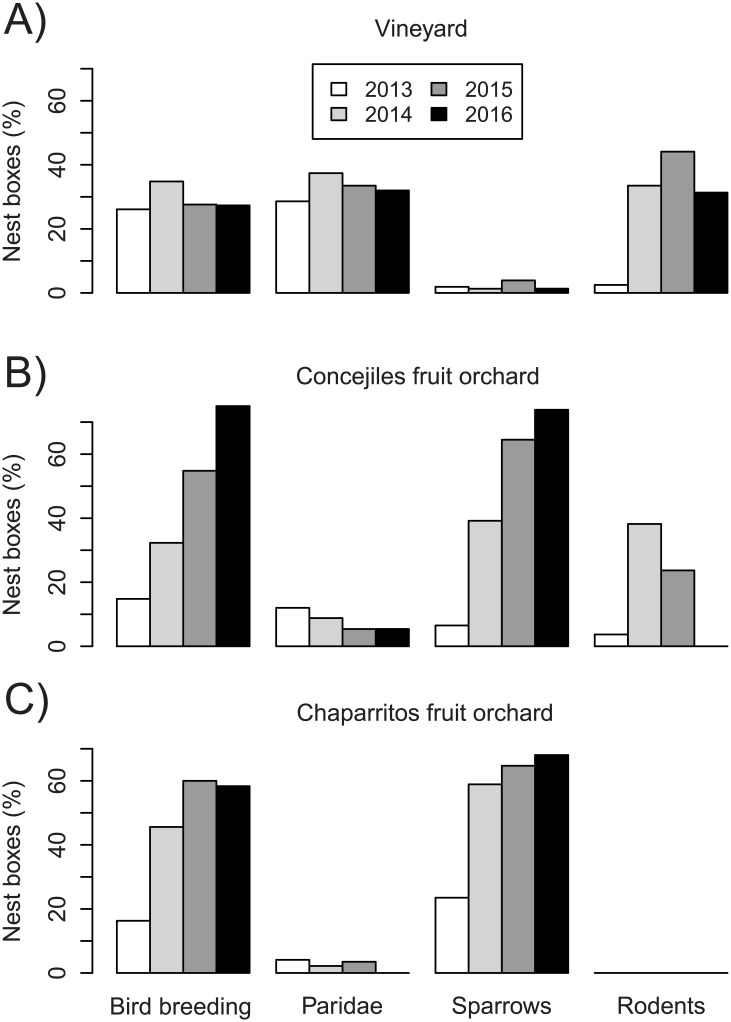
Relevant categories of use for nest boxes installed in the (A) Abadía Retuerta vineyard, (B) Concejiles fruit orchard and (C) Chaparrito fruit orchard, across the four years of study. Bird breeding refers to verified hatched eggs or recruited fledglings (categories 4–5 of nest box use, see Methods). Bars for insectivorous birds (*Paridae*) and sparrows refer to categories 2 (finished nest) to 5 (recruited fledglings). Nearly all observations contributing to the “Rodents” bar refer to Garden dormouse (category 6).

The following species nested in the installed boxes: Great tit, Blue tit, Coal tit, Crested tit, House sparrow (*Passer domesticus)*, Eurasian tree sparrow (*Passer montanus)* and, importantly, Garden dormouse (*Eliomys quercinus*). The number of nest boxes containing birds with verified reproductive events, i.e. hatched eggs or recruited fledglings, averaged 53.3% (range was 27.3–75.0%) across the three field sites by the fourth year (Fig 2). In the same year, occupancy by birds that had finished a nest and laid eggs averaged 6.7% of nest boxes (range was 4.3–9.8%; S2 Table) in addition to those with verified reproductive events.

Great tit was by far the insectivorous bird species that most often used the nest boxes, accounting for 161 of the 197 (81.73%) reproductive events at the three sites over the whole studied period. Use of nest boxes by sparrows was remarkably high at the two fruit orchards, where use by insectivorous birds was negligible (Fig 2B and 2C). Use of nest boxes by Garden dormouse was remarkably high in the vineyard: it exceeded >30% in years 2–4 (Fig 2A). Leaving nest boxes uncovered before the breeding season in the fourth year reduced occupancy by this rodent from 44% to 31% in the vineyard and from 24% to 0% in the Concejiles fruit orchard.

### Explanatory factors of nest box use and breeding

Spearman’s rank correlations of category of use across nest boxes between two consecutive years (H1) were positive and significant: p values for the three year-pairings were <0.01 for the Abadía Retuerta vineyard, <0.02 for the Concejiles fruit orchard, and <0.0001 for the Chaparrito fruit orchard. (The only exception was p = 0.67 for the Concejiles fruit orchard for the 2015–2016 correlation.) These positive correlations indicate growing use of the same nest boxes by birds for nesting and breeding year after year.

**Fig 3:**
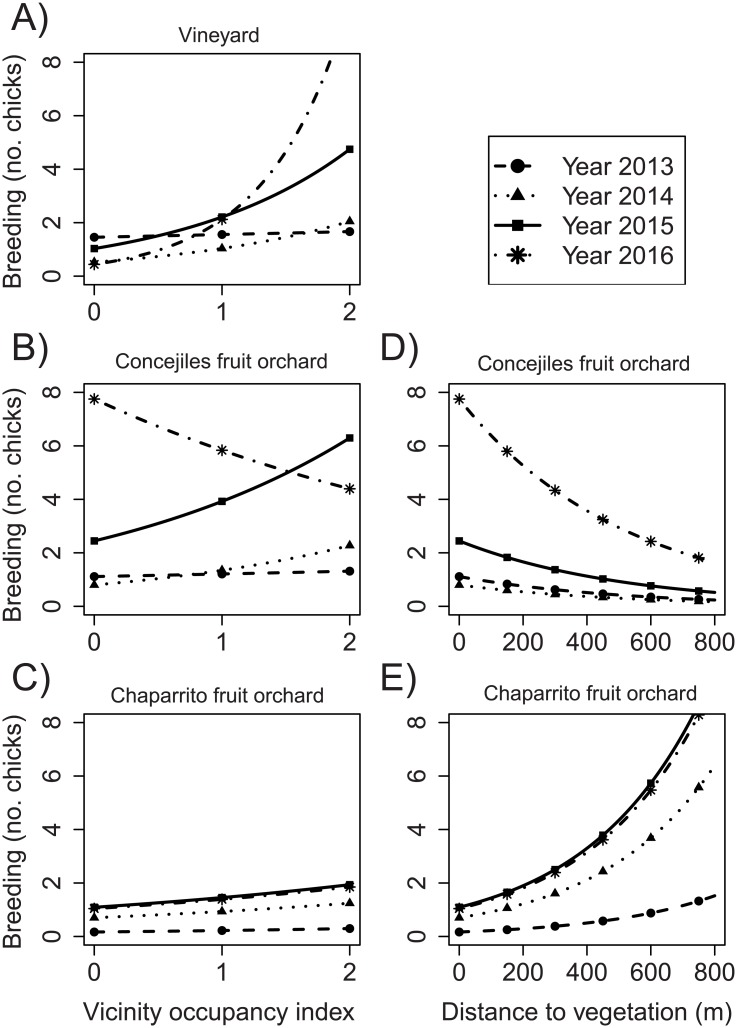
Model-averaged estimated effects of (A-C) occupancy by birds of adjacent nest boxes and (D-E) distance to natural or semi-natural vegetation on bird breeding at the (A) vineyard, (B, D) Concejiles fruit orchard, and (C, E) Chaparrito fruit orchard, across the four years of study. Breeding was measured as the number of laid eggs or chicks/fledglings that were counted at a nest box. A mean distance to natural or semi-natural vegetation of 400 m was used to generate model predictions in panels B and C. Zero occupancy of adjacent nest boxes was used to generate model predictions in panels D and E. Distance to natural or semi-natural vegetation did not apply to the vineyard.

GLMMs to analyze bird breeding (H2 and H3), measured as the number of laid eggs or chicks/fledglings that were counted at a nest box, resulted in one single best model for the vineyard and four best models for each of the tree orchards (Table 1). For the three sites, bird breeding was affected simultaneously by year and occupancy of adjacent nest boxes and, in the case of the fruit orchards, also by distance to natural or semi-natural vegetation (Table 1). There was a trend to more breeding year after year, and this was remarkably strong in the fruit orchards (Fig 3A–3E, S3 Table). The effect of occupancy of adjacent nest boxes was positive at all three sites and in all years, and it was particularly strong in the vineyard (Fig 3A–3C, S3 Table). The effect of distance to natural or semi-natural vegetation was weak and negative at the Concejiles fruit orchard and positive at the Chaparrito fruit orchard; the effect intensified with time at both sites (Fig 3D and 3E, S3 Table).

**Table 1:** AIC_c_-based comparison of alternative models of bird breeding at the Abadía Retuerta vineyard and Concejiles and Chaparrito fruit orchards, as a function of year (Yr), distance to natural or semi-natural vegetation (Dist), and occupancy of adjacent nest boxes (OcAN). Pairwise interactions of these variables were also included (*). The null model included only a constant term: the mean of observed values. The best models for Abadía Retuerta had no random effects, whereas models for Concejiles and Chaparrito included a random effect of nest box on the intercept. The best models (lowest AIC_c_) are indicated in boldface type. The indicated number of parameters describes the best model, or the most complex model when there were multiple best models.

Fixed-effect models	AIC_c_		
	Abadía Retuerta	Concejiles	Chaparrito
Null model (no effects)	2924.5	1218.4	1101.1
Yr	2875.0	1182.1	**1069.0**
Dist	-	1213.5	1101.4
OcAN	1563.9	1189.8	1103.1
Yr + Dist	-	1173.5	**1068.8**
Yr * Dist	-	1176.4	1072.5
Yr + OcAN	1563.6	1165.7	1069.4
Yr * OcAN	**1560.3**	1164.4	1075.1
Dist + OcAN	-	1189.6	1103.2
Dist * OcAN	-	1189.9	1105.0
Yr + Dist + OcAN	-	**1162.9**	**1068.4**
Yr * Dist + OcAN	-	1165.9	1072.0
Yr * OcAN + Dist	-	**1161.5**	1074.4
Yr + OcAN*Dist	-	**1163.4**	**1067.2**
Yr * Dist + Yr * OcAN	-	1166.5	1077.6
Yr * Dist + Dist * OcAN	-	1165.7	1070.9
Yr * OcAN + Dist * OcAN	-	**1161.9**	1073.3
Year * Dist + Year * OcAN + Dist * OcAN	-	1166.7	1077.0
**Number of parameters**	**10**	**12**	**9**

### Predation of sentinels

**Fig 4:**
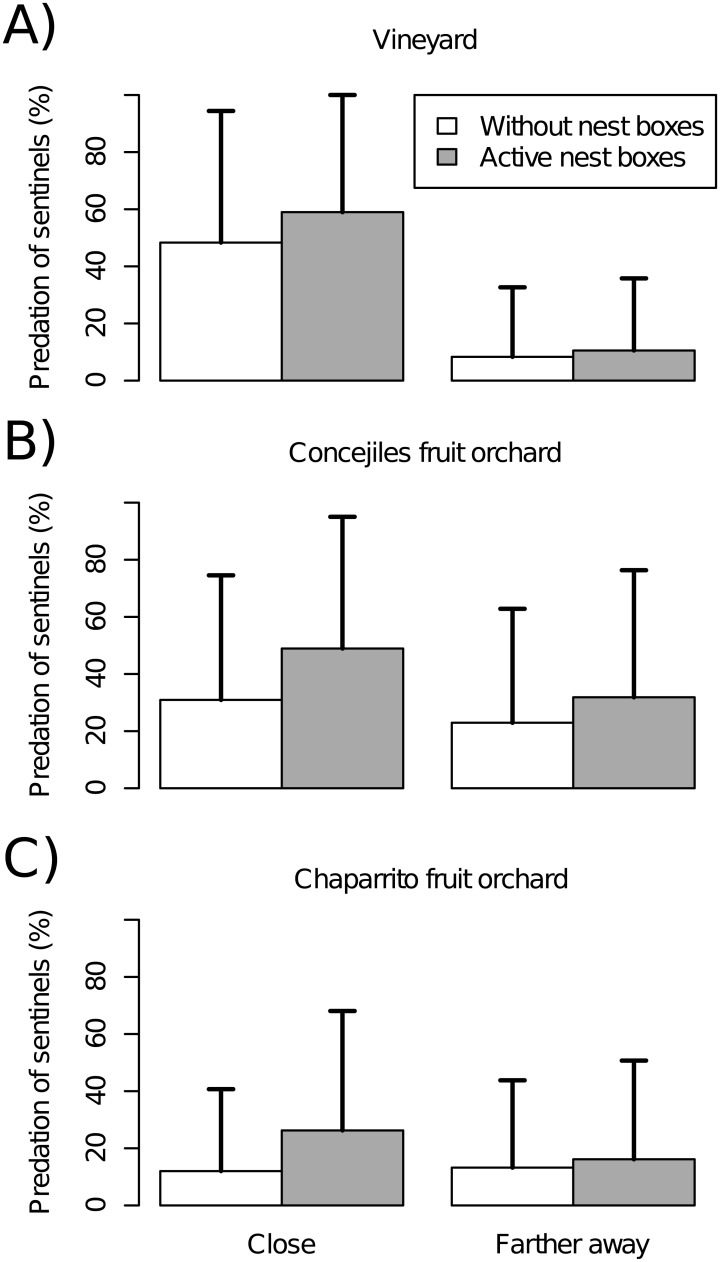
Predation of sentinel caterpillars samples closer to, or farther away from, active nest boxes and their corresponding paired samples in distant areas without nest boxes at (A) the vineyard, (B) Concejiles fruit orchard and (C) Chaparrito fruit orchard, across the four years of study. In the vineyard, the sample closer to the active nest box lay <6 m from the box and the sample farther from the active nest box lay >15 m away from the box. In the fruit orchards, the sample closer to the active nest box was 3 m away and the sample farther from the active nest box was 10 m from the box.

Predation of sentinel caterpillars at the three field sites was affected by treatment (i.e. presence of active nest boxes vs. distant area without nest boxes), proximity of sentinel samples to active nest boxes (closer vs. farther), and the interaction between these two factors (Table 2). In the vineyard, there were two best-fit models, one with and another without the interaction term (Table 2). Overall, predation rates at the three field sites over the whole study period were approximately one third higher in active nest box samples (31.51 ± 43.13%) than in distant areas without nest boxes (22.45 ± 38.58%; S4 Table). Similarly, the overall level of predation of sentinel caterpillars was higher for samples closer to active nest boxes within the treatment areas than for samples farther away from the boxes, except at the Chaparrito site (Fig 4, S5 Table). Goodness of fit based on marginal and conditional R^2^ values was intermediate for the vineyard, low for the Concejiles fruit orchard, and negligible for the Chaparrito fruit orchard (Table 2).

**Table 2:** AIC_c_-based comparison of alternative models of predation of sentinel caterpillar samples (%) at the Abadía Retuerta vineyard and the Concejiles and Chaparrito fruit orchards, as a function of Treatment (active nest boxes vs. control sites) and Proximity to active nest boxes (close vs. farther away samples). Interaction of these two variables (*) was also included. The null model included only a constant term: the mean of observed values. All models, including the null model, had the same random structure, representing the effect of nest box on the intercept. The best model (lowest AIC_c_) is indicated in boldface type. The number of parameters, conditional R^2^ (fixed and random effects) and marginal R^2^ (only fixed effects) refer to the best model.

Fixed-effect models	AIC_c_		
	Abadía Retuerta	Concejiles	Chaparrito
Null model (no effects)	4269.4	2810.3	2500.1
Treatment	4247.5	2732.0	2435.4
Proximity	3011.5	2742.8	2484.4
Treatment + Proximity	**2979.1**	2662.2	2419.2
Treatment * Proximity	**2979.1**	**2658.0**	**2397.9**
**Number of parameters**	**6**	**6**	**6**
**R**^**2**^**m**	**0.317**	**0.178**	**6.487·e**^**-04**^
**R**^**2**^**c**	**0.417**	**0.208**	**6.496·e**^**-04**^

### Food consumption by insectivorous birds

Estimated mean food consumption rates per ha and breeding season by insectivorous birds ranged from 0.02 kg in the Chaparrito fruit orchard–where insectivorous birds hardly nested- to 0.15 kg in the vineyard (Table 3). Calculations in terms of the number of breeding pairs and average number of chicks of the different bird species for every field site and year can be found in S6 Table.

**Table 3:** Estimated food consumption, expressed in kg per breeding season (b.s.) or kg ha^-1^ per breeding season, for insectivorous species and field sites. Values shown are the mean ± standard deviation at each field site across the four breeding seasons.

Field site	Great tit (kg b.s.^-1^)	Blue tit (kg b.s.^-1^)	Coal tit (kg b.s.^-1^)	Crested tit (kg b.s.^-1^)	All species (kg ha^-1^b.s.^-1^)
Abadía Retuerta vineyard	24.87 ± 2.43	4.36 ± 0.42	0.47 ± 0.37	0.54 ± 0.66	0.15 ±0.01
Concejiles fruit orchard	5.30 ± 3.05	0.18 ± 0.37	0	0	0.09 ± 0.04
Chaparrito fruit orchard	1.65 ± 1.11	0.31 ± 0.63	0	0	0.02 ± 0.02

## Discussion

We assessed the potential of insectivorous birds to act as natural enemies of pests in a variety of Mediterranean woody crops in central Spain. By monitoring installed nest boxes, measuring predation rates using sentinel caterpillars, and estimating their food consumption over four consecutive years, we obtained further evidence that these species can help regulate pests and provide farmers with useful learned lessons for a more friendly agricultural practice.

### Use of nest boxes

Our first objective was to evaluate nest box use by insectivorous birds and other species, with a focus on bird breeding. Occupancy by insectivorous birds was null in our preliminary trial at the vineyard, meaning that resident avian species may have preferred nest boxes that were covered by vegetation [34]. We hypothesize that the relatively low number of bird breeding events at the installed nest boxes in the vineyard (only 34% by the fourth year) may reflect the abundance of natural nesting sites in the surrounding Mediterranean woodland and riparian forest [46, 47], as well as the abundance of Garden dormouse. In contrast, the number of breeding events at nest boxes in the fruit orchards was moderate (≥50%) and consistent with previous studies [e.g. 48–50, 49].

Other animal species besides the target insectivorous birds used the installed nest boxes. Sparrows, favored by the agricultural habitat (herbaceous cropland and pastures) and associated farm buildings around the fruit orchards [51], competed against insectivorous birds for nesting sites at these woody crops in our study. Nevertheless, these omnivorous species may still contribute to pest regulation as they eat many invertebrates during the breeding season [52]. In addition, sparrow populations are declining in Europe [53], and installation of nest boxes in woody crops may help to reverse this trend. Use of nest boxes by Garden dormouse was remarkably high at the vineyard because the surrounding forest habitat, particularly the pine plantations, favors this rodent, which preys upon eggs and chicks in nests [54–56]. This result suggests that nest boxes should be designed to prevent or minimize occupancy by dormice [57, 58]. One practical solution, as this study shows, may be leave the nest boxes uncovered before the breeding season.

Our second objective was to evaluate the factors that determine breeding of insectivorous birds at the nest boxes. As hypothesized (H1), we found that use of nest boxes by birds increased with time at the three field sites; this fact may reflect learning by birds, increase in local bird populations, and weather effects [59, 60]. We also found evidence of habitat selection by birds (H2) at the level of both micro-habitat (nest box) and meso-habitat (patch). Thus, habitat quality seems to exert a stronger effect than competition on site selection for bird breeding, leading to bird reproductive events in the same nest boxes year after year and aggregation of breeding pairs in high-quality patches in the same year [58, 61, 62]. The hypothesized higher breeding close to natural or semi-natural vegetation (H3) could be tested only in fruit orchards. Results at the Concejiles fruit orchard were consistent with this hypothesis, probably reflecting the large riparian forest adjacent to this site that acted as source habitat [47, 63]. However, the results at the Chaparrito fruit orchard were counter to the hypothesis, probably reflecting low habitat quality of short pear trees close to degraded riparian vegetation.

### Predation and food consumption by insectivorous birds

We found higher predation of sentinel caterpillars close to active nest boxes than at distant areas without nest boxes (objective 3), in accordance with H4 and previous studies [21, 64]. This indicates a strong “cleaning” effect close to bird nests that weakens with increasing distance from nests [19], as distances of farther away samples were not outside the feeding range of the target species. The sentinel experiments come with two caveats. One is that caterpillars were fixed to tree twigs, while sparrows forage primarily on the ground [65]. The other is that the caterpillars were not consistently monitored by camera traps or direct observation, meaning that we cannot exclude that they were consumed by birds other than those nesting in the boxes or, much less likely, by other animals such as rodents.

Our fourth objective was to estimate food consumption by insectivorous birds, which in our study ranged from 0.02 to 0.15 kg ha^-1^ per breeding season, the lowest value of the range corresponding to a fruit orchard with neglible nesting of insectivorous birds. These values are not as high as in previous studies [20–23, 66, 67]. For instance, in Californian vineyard, an adult pair of Western bluebirds consumed an average of 46 g of arthropod prey per day in one study, and an average five-chick cluster consumed 78 g of arthropod prey per day [66]. Insectivorous farmland birds have been estimated to consume at least 1,000 kg pest invertebrates km^-2^ yr^-1^ in Kenya [67]. We have underestimated food consumption at our study sites, since our estimates (a) refer only to the breeding season and not the entire year, (b) refer to insectivorous birds (that are sedentary in the study area) but not sparrows, and (c) do not include consumption by brood of breeding birds with unverified hatched eggs. We acknowledge as well that reference data for adult consumption of Great tit [35] are from captive birds that may not reflect behaviour of wild birds, and that Blue tits, Coal tits and Crested tits show different foraging behaviour and have species-specific preferences than Great tit that were not taken into account. On the other hand, estimates of food consumption by insectivorous birds include not only consumption of pest arthropods, but also predation of beneficial arthropods such as spiders [16, 17, 63, 68–71]. Nevertheless, our estimated food consumption demonstrates the substantial potential of insectivorous birds to regulate pests as an alternative to pesticides.

## Conclusions and perspectives

In our study, bird breeding increased at nest boxes from one year to the next, and habitat characteristics strongly influenced selection of nest boxes and breeding. However, the distance to natural or semi-natural vegetation did not consistently affect bird breeding. Predation of sentinel caterpillars was significantly higher close to active nest boxes than at paired distant areas without nest boxes; predation declined rapidly with increasing distance from the nest box. Food consumption by insectivorous birds was conservatively estimated to average at least 0.02 kg ha^-1^ per breeding season. We conclude that installation of nest boxes in Mediterranean woody crops enhances populations of insectivorous birds that regulate pests and can reverse population decline of some farmland bird species. However, the magnitude of potential of pest regulation is moderate and highly dependent on context [72], particularly the composition and configuration of landscape around the target crops, as well as the activity of species such as Garden dormouse. The effects of insectivorous birds on the entire arthropod community, including on species beneficial for pest regulation, need to be elucidated. Future work should verify our estimates of food consumption by insectivorous birds in Mediterranean woody crops. Our results suggest that installation of nest boxes by itself cannot replace the effects of conventional control of arthropod pests and may need to be used together with other habitat manipulations that positively contribute to conservation biological control. We suggest that restoration of natural and semi-natural vegetation in homogenous agricultural landscapes in temperate areas, such as planting forest islands and hedgerows, can enhance pest regulation by insectivorous birds [12, 73, 74]. We expect that the results of this study and related work will encourage farm managers to install nest boxes as a way to reduce or eliminate pesticide use.

## Supporting information

S1 Table Mean temperature and precipitation at the studied vineyard and fruit orchards between February and June in 2012–2016. Except for a preliminary trial at the vineyard, all field work was carried out from 2013 to 2016, and nest box exploration, occupancy and breeding by insectivorous birds occurred between February and July each year. Data are not reported for the two olive groves included in the study because nest box occupancy by birds was found to be null at those sites throughout the four-year study.
(DOC)

S2 Table Use of nest boxes at each field site and year. Category 6 refers mostly to rodents, particularly Garden dormouse.
(DOC)

S3 Table Model-averaged estimates, standard errors (Std. Error) and relative importance (wi) of selected variables in the best models of bird breeding at the Abadía Retuerta vineyard and the Concejiles and Chaparrito fruit tree orchards. The intercept summarizes the level in 2013. Estimated variance and standard deviation (Std. Dev) are shown for random effects. Estimated coefficients refer to the response variable on a log scale. Yr, Year; Dist, Distance to natural or semi-natural vegetation; OcAN, Occupancy of adjacent nest boxes (OcAN).
(DOC)

S4 Table Predation of sentinel samples of Greater wax moth (Galleria mellonella) caterpillars close to, or farther away from, active nest boxes and of paired sentinel samples in distant areas without nest boxes. Values shown are percentage means ± standard deviations.
(DOC)

S5 Table Model-averaged estimates and standard errors (Std. Error) of the best models of predation of sentinel caterpillar samples (%) at the Abadía Retuerta vineyard and the Concejiles and Chaparrito fruit tree orchards. The intercept summarizes the levels close to active nest boxes and distant areas without nest boxes. Estimated variance and standard deviation (Std. Dev) are shown for random effects. For simplicity, only the most complex model is shown for Abadía Retuerta (i.e. no model averaging was performed), although the two best models had similar support (Table 2). Estimated coefficients refer to the response variable on a logit scale.
(DOC)

S6 Table Disaggregated estimates of food consumption (grams) by insectivorous birds during the breeding season, based on published consumption rates for breeding Great tit (Parus major), weight of the other breeding insectivorous bird species relative to the weight of Great tit, number of breeding pairs and number of chicks per breeding pair in the studied field sites and the four years of study. (DOC)
