# Supplementary material for: Potential of pest regulation by insectivorous birds in Mediterranean woody crops
Source: PLoS One. 2017 Sep 6;12(9):e0180702. doi: 10.1371/journal.pone.0180702 (PMC5587304; doi:10.1371/journal.pone.0180702)
Supplement: S1 Table — Except for a preliminary trial at the vineyard, all field work was carried out from 2013 to 2016, and nest box exploration, occupancy and breeding by insectivorous birds occurred between February and July each year. Data are not reported for the two olive groves included in the study because nest box occupancy by birds was found to be null at those sites throughout the four-year study. (DOC) [file pone.0180702.s001.doc]

**S1 Table.** Mean temperature and precipitation at the studied vineyard and fruit orchards between February and June in 2012-2016. Except for a preliminary trial at the vineyard, all field work was carried out from 2013 to 2016, and nest box exploration, occupancy and breeding by insectivorous birds occurred between February and July each year. Data are not reported for the two olive groves included in the study because nest box occupancy by birds was found to be null at those sites throughout the four-year study.

|  | **Abadía Retuerta** | | **Concejiles** | | **Chaparrito** | |
| --- | --- | --- | --- | --- | --- | --- |
|  | **Mean Temp. (ºC)** | **Precip. (mm)** | **Mean Temp. (ºC)** | **Precip. (mm)** | **Mean Temp. (ºC)** | **Precip. (mm)** |
| **2012** | 10,5 | 175 | 15,5 | 85 | 14,6 | 84 |
| **2013** | 9,1 | 281 | 14,7 | 303 | 14,5 | 311 |
| **2014** | 11,6 | 169 | 16,1 | 146 | 15,7 | 250 |
| **2015** | 11,6 | 157 | 17,1 | 107 | 16,3 | 132 |
| **2016** | 10.1 | 236 | 15.2 | 228 | 14.5 | 275 |
