# Supplementary material for: Potential of pest regulation by insectivorous birds in Mediterranean woody crops
Source: PLoS One. 2017 Sep 6;12(9):e0180702. doi: 10.1371/journal.pone.0180702 (PMC5587304; doi:10.1371/journal.pone.0180702)
Supplement: S2 Table — Category 6 refers mostly to rodents, particularly Garden dormouse. (DOC) [file pone.0180702.s002.doc]

**S2 Table. Use of nest boxes at each field site and year. Category 6 refers mostly to rodents, particularly Garden dormouse.**

| **Abadía Retuerta vineyard** | | | |  |
| --- | --- | --- | --- | --- |
|  | **No. (%) nest boxes** | | | |
| **Category of use** | **2013** | **2014** | **2015** | **2016** |
| **0. Empty nest box** | 63 (39) | 20 (13) | 10 (7) | 30 (20) |
| **1. Initiated but unfinished bird nest** | 45 (28) | 22 (14) | 18 (12) | 14 (9) |
| **2. Finished bird nest** | 2 (1) | 4 (3) | 4 (3) | 1 (1) |
| **3. Laid eggs** | 5 (3) | 3 (2) | 11 (7) | 9 (6) |
| **4. Hatched eggs** | 3 (2) | 2 (1) | 2 (1) | 1 (1) |
| **5. Recruited fledglings** | 39 (24) | 52 (34) | 40 (26) | 40 (27) |
| **6. Presence of species other than birds** | 4 (2) | 52 (34) | 67 (44) | 55 (37) |
| **Concejiles fruit orchard** | | | | |
|  | **2013** | **2014** | **2015** | **2016** |
| **0. Empty nest box** | 41 (38) | 4 (4) | 0 (0) | 2 (2) |
| **1. Initiated but unfinished bird nest** | 42 (39) | 10 (10) | 6 (6) | 20 (22) |
| **2. Finished bird nest** | 4 (4) | 10 (10) | 3 (3) | 2 (2) |
| **3. Laid eggs** | 1 (1) | 6 (6) | 11 (12) | 5 (5) |
| **4. Hatched eggs** | 1 (1) | 2 (2) | 4 (4) | 10 (11) |
| **5. Recruited fledglings** | 15 (14) | 31 (30) | 47 (51) | 53 (58) |
| **6. Presence of species other than birds** | 4 (4) | 39 (38) | 22(24) | 0 (0) |
| **Chaparrito fruit orchard** | | | |  |
|  | **2013** | **2014** | **2015** | **2016** |
| **0. Empty nest box** | 14 (14) | 3 (3) | 1 (1) | 0 (0) |
| **1. Initiated but unfinished bird nest** | 57 (58) | 32 (36) | 26 (31) | 22 (31) |
| **2. Finished bird nest** | 10 (10) | 12 (13) | 2 (2) | 1 (1) |
| **3. Laid eggs** | 1 (1) | 2 (2) | 5 (6) | 6 (8) |
| **4. Hatched eggs** | 0 (0) | 0 (0) | 0 (0) | 0 (0) |
| **5. Recruited fledglings** | 16 (16) | 41 (46) | 51 (60) | 43 (60) |
| **6. Presence of species other than birds** | 0 (0) | 0 (0) | 0 (0) | 0 (0) |
