# Supplementary material for: Potential of pest regulation by insectivorous birds in Mediterranean woody crops
Source: PLoS One. 2017 Sep 6;12(9):e0180702. doi: 10.1371/journal.pone.0180702 (PMC5587304; doi:10.1371/journal.pone.0180702)
Supplement: S3 Table — The intercept summarizes the level in 2013. Estimated variance and standard deviation (Std. Dev) are shown for random effects. Estimated coefficients refer to the response variable on a log scale. Yr, Year; Dist, Distance to natural or semi-natural vegetation; OcAN, Occupancy of adjacent nest boxes (OcAN). (DOC) [file pone.0180702.s003.doc]

**S3 Table.** Model-averaged estimates, standard errors (Std. Error) and relative importance (w_i_) of selected variables in the best models of bird breeding at the Abadía Retuerta vineyard and the Concejiles and Chaparrito fruit tree orchards. The intercept summarizes the level in 2013. Estimated variance and standard deviation (Std. Dev) are shown for random effects. Estimated coefficients refer to the response variable on a log scale. Yr, Year; Dist, Distance to natural or semi-natural vegetation; OcAN, Occupancy of adjacent nest boxes (OcAN).

| **Abadía Retuerta vineyard** |  |  |  |
| --- | --- | --- | --- |
| *Random effects* | *Variance* | *Std. Dev.* |  |
| Nest box (intercept) | - | 2.980 |  |
| Nest box (OcAN) | - | 2.432 |  |
| *Fixed effects* | *Estimate* | *Std. Error* | *w_i_* |
| Intercept | 0.375 | 0.264 | 1.00 |
| OcAN | 0.068 | 0.315 | 1.00 |
| Yr(2014) | -1.022 | 0.414 | 1.00 |
| Yr(2015) | -0.344 | 0.382 | 1.00 |
| Yr(2016) | -1.195 | 0.394 | 1.00 |
| OcAN:Yr(2014) | 0.615 | 0.451 | 1.00 |
| OcAN:Yr(2015) | 0.695 | 0.419 | 1.00 |
| OcAN:Yr(2016) | 1.505 | 0.430 | 1.00 |
| **Concejiles fruit orchard** |  |  |  |
| *Random effects* | *Variance* | *Std. Dev.* |  |
| Nest box (intercept) | 0.269 | 0.519 |  |
| *Fixed effects* | *Estimate* | *Std. Error* | *w_i_* |
| Intercept | -0.109 | 0.423 | 1.00 |
| Dist | -1.486·e^-03^ | 7.513·e^-04^ | 1.00 |
| OcAN | 0.289 | 0.410 | 1.00 |
| Yr(2014) | -0.218 | 0.419 | 1.00 |
| Yr(2015) | 0.889 | 0.437 | 1.00 |
| Yr(2016) | 1.761 | 0.450 | 1.00 |
| Dist:Yr(2014) | 0.440 | 0.482 | 067 |
| Dist:Yr(2015) | 0.417 | 0.476 | 0.67 |
| Dist:Yr(2016) | -0.351 | 0.462 | 0.67 |
| Dist:OcAN | 7.389·e^-04^ | 5.679·e^-04^ | 0.44 |
| **Chaparrito fruit orchard** |  |  |  |
| *Random effects* | *Variance* | *Std. Dev.* |  |
| Nest box (intercept) | 1.001 | 1.001 |  |
| *Fixed effects* | *Estimate* | *Std. Error* | *w_i_* |
| Intercept | -1.395 | 0.567 | 1.00 |
| Dist | 1.989·e^-03^ | 1.223·e^-03^ | 0.83 |
| OcAN | 0.101 | 0.373 | 0.65 |
| Yr(2014) | 1.364 | 0.282 | 1.00 |
| Yr(2015) | 1.790 | 0.290 | 1.00 |
| Yr(2016) | 1.787 | 0.277 | 1.00 |
| Dist:OcAN | 3.274·e^-04^ | 5.269·e^-04^ | 0.42 |
