# Supplementary material for: Potential of pest regulation by insectivorous birds in Mediterranean woody crops
Source: PLoS One. 2017 Sep 6;12(9):e0180702. doi: 10.1371/journal.pone.0180702 (PMC5587304; doi:10.1371/journal.pone.0180702)
Supplement: S4 Table — Values shown are percentage means ± standard deviations. (DOC) [file pone.0180702.s004.doc]

**S4 Table.** Predation of sentinel samples of Greater wax moth (*Galleria mellonella*) caterpillars close to, or farther away from, active nest boxes and of paired sentinel samples in distant areas without nest boxes. Values shown are percentage means ± standard deviations.

| **A. Retuerta vineyard** | Active nest boxes − Close | Without  nest boxes | Active nest boxes − Farther away | Without  nest boxes |
| --- | --- | --- | --- | --- |
| 2013 | 64.71±43.50 | 46.76±48.60 | 14.71±29.46 | 11.76±32.70 |
| 2014 | 59.17±46.43 | 59.17±39.11 | 7.92±22.26 | 9.58±19.89 |
| 2015 | 51.85±45.74 | 37.04±46.14 | 7.04±19.77 | 0.74±2.67 |
| 2016 | 58.70±43.93 | 52.61±48.54 | 11.30±27.68 | 10.87±27.45 |
| **Concejiles fruit orchard** |  |  |  |  |
| 2013 | 50.00±46.58 | 49.29±49.84 | 30.00±46.24 | 47.86±48.70 |
| 2014 | 52.63±42.67 | 36.84±45.71 | 25.79±40.18 | 26.32±44.12 |
| 2015 | 45.83±48.54 | 27.92±43.14 | 28.75±42.97 | 7.08±20.74 |
| 2016 | 48.33±49.50 | 14.44±32.22 | 43.89±50.54 | 21.11±39.84 |
| **Chaparrito fruit orchard** |  |  |  |  |
| 2013 | 13.60±32.39 | 2.00±8.16 | 12.80±32.98 | 8.40±25.11 |
| 2014 | 20.69±38.45 | 5.86±15.47 | 8.97±24.25 | 13.79±30.05 |
| 2015 | 47.50±47.39 | 22.50±40.46 | 29.58±43.09 | 22.08±40.00 |
| 2016 | 24.76±43.20 | 20.48±36.67 | 14.76±35.72 | 8.10±23.79 |
