# Supplementary material for: Potential of pest regulation by insectivorous birds in Mediterranean woody crops
Source: PLoS One. 2017 Sep 6;12(9):e0180702. doi: 10.1371/journal.pone.0180702 (PMC5587304; doi:10.1371/journal.pone.0180702)
Supplement: S5 Table — The intercept summarizes the levels close to active nest boxes and distant areas without nest boxes. Estimated variance and standard deviation (Std. Dev) are shown for random effects. For simplicity, only the most complex model is shown for Abadía Retuerta (i.e. no model averaging was performed), although the two best models had similar support (Table 2). Estimated coefficients refer to the response variable on a logit scale. (DOC) [file pone.0180702.s005.doc]

**S5 Table. Model-averaged estimates and standard errors (Std. Error) of the best models of predation of sentinel caterpillar samples (%) at the Abadía Retuerta vineyard and the Concejiles and Chaparrito fruit tree orchards. The intercept summarizes the levels close to active nest boxes and distant areas without nest boxes. Estimated variance and standard deviation (Std. Dev) are shown for random effects. For simplicity, only the most complex model is shown for Abadía Retuerta (i.e. no model averaging was performed), although the two best models had similar support (Table 2). Estimated coefficients refer to the response variable on a logit scale.**

| **Abadía Retuerta** |  |  |
| --- | --- | --- |
| *Random effects* | *Variance* | *Std. Dev.* |
| Nest box | 2.347 | 1.532 |
| *Fixed effects* | *Estimate* | *Std. Error* |
| Intercept | -0.227 | 0.206 |
| Treatment | 0.573 | 0.100 |
| Proximity (Farther away) | -2.913 | 0.145 |
| Treatment : Proximity (Farther away) | -0.268 | 0.189 |
| **Concejiles** |  |  |
| *Random effects* | *Variance* | *Std. Dev.* |
| Nest box | 3.801 | 1.950 |
| *Fixed effects* | *Estimate* | *Std. Error* |
| Intercept | -1.652 | 0.299 |
| Treatment | 1.043 | 0.126 |
| Proximity (Farther away) | -0.528 | 0.133 |
| Treatment : Proximity (Farther away) | -0.458 | 0.182 |
| **Chaparrito** |  |  |
| *Random effects* | *Variance* | *Std. Dev.* |
| Nest box | 4.227 | 2.056 |
| *Fixed effects* | *Estimate* | *Std. Error* |
| Intercept | -3.036 | 0.324 |
| Treatment | 1.253 | 0.138 |
| Proximity (Farther away) | 0.135 | 0.148 |
| Treatment : Proximity (Farther away) | -0.959 | 0.197 |
