# Supplementary material for: Potential of pest regulation by insectivorous birds in Mediterranean woody crops
Source: PLoS One. 2017 Sep 6;12(9):e0180702. doi: 10.1371/journal.pone.0180702 (PMC5587304; doi:10.1371/journal.pone.0180702)
Supplement: S6 Table — (DOC) [file pone.0180702.s006.doc]

**S6 Table. Disaggregated estimates of food consumption (grams) by insectivorous birds during the breeding season, based on published consumption rates for breeding Great tit (*Parus major*), weight of the other breeding insectivorous bird species relative to the weight of Great tit*,* number of breeding pairs and number of chicks per breeding pair in the studied field sites and the four years of study.**

|  | **Great tit**  **Breeding pairs/Avg. # chicks/**  **Estimated consumption by adults/chicks/juveniles** | **Blue tit**  **Breeding pairs/Avg. # chicks**  **Estimated consumption by adults/chicks/juveniles** | **Coal tit**  **Breeding pairs/Avg. # chicks**  **Estimated consumption by adults/chicks/juveniles** | **Crested tit**  **Breeding pairs/Avg. # chicks**  **Estimated consumption by adults/chicks/juveniles** |
| --- | --- | --- | --- | --- |
| **Abadía Retuerta vineyard** | | | | |
| **2013** | 30.00 / 5.35 / 22,644.00 / 66.07 / 504.77 | 8.00 / 6.00 / 3,441.92 / 42.24 / 322.68 | 1.00 / 7.00 / 354.38 / 40.63 / 310.41 | 2.00 / 7.00 / 890.64 / 51.01 / 389.67 |
| **2014** | 31.00 / 4.68 / 23,398.80 / 57.80 / 441.56 | 10.00 / 6.33 / 4,302.40 / 44.56 / 340.42 | 0 / 0 / 0 / 0 / 0 | 0 / 0 / 0 / 0 / 0 |
| **2015** | 37.00 / 5.22 / 27,927.60 / 64.47 / 492.51 | 9.00 / 6.62 / 3,872.16 / 46.60 / 356.02 | 1.00 / 9.00 / 354.38 / 52.24 / 399.10 | 0 / 0 / 0 / 0 / 0 |
| **2016** | 31.00 / 4.44 / 23,398.80 / 54.83 / 418.91 | 10.00 / 6.29 / 4,302.40 / 44.28 / 338.27 | 1.00 / 0 / 354.38 / 0 / 0 | 1.00 / 6.00 / 445.32 / 43.72 / 333.99 |
| **Concejiles fruit tree orchard** | | | | |
| **2013** | 11.00 / 6.73 / 8,302.80 / 83.12 / 634.98 | 1.00 / 5.00 / 430.24 / 35.20 / 268.90 | 0 / 0 / 0 / 0 / 0 | 0 / 0 / 0 / 0 / 0 |
| **2014** | 7.00 / 7.00 / 5,283.60 / 86.45 / 660.45 | 0 / 0 / 0 / 0 / 0 | 0 / 0 / 0 / 0 / 0 | 0 / 0 / 0 / 0 / 0 |
| **2015** | 5.00 / 6.20 / 3,774 / 76.57 / 584.97 | 0 / 0 / 0 / 0 / 0 | 0 / 0 / 0 / 0 / 0 | 0 / 0 / 0 / 0 / 0 |
| **2016** | 2.00 / 2.00 / 1,509.60 / 24.70 / 188.70 | 0 / 0 / 0 / 0 / 0 | 0 / 0 / 0 / 0 / 0 | 0 / 0 / 0 / 0 / 0 |
| **Chaparrito fruit tree orchard** | | | | |
| **2013** | 2.00 / 5.50 / 1,509.60 / 67.93 / 518.93 | 2.00 / 6.50 / 860.48 / 45.76 / 349.57 | 0 / 0 / 0 / 0 / 0 | 0 / 0 / 0 / 0 / 0 |
| **2014** | 2.00 / 6.00 / 1,509.60 / 74.10 / 566.10 | 0 / 0 / 0 / 0 / 0 | 0 / 0 / 0 / 0 / 0 | 0 / 0 / 0 / 0 / 0 |
| **2015** | 3.00 / 8.00 / 2,264.40 / 98.80 / 754.80 | 0 / 0 / 0 / 0 / 0 | 0 / 0 / 0 / 0 / 0 | 0 / 0 / 0 / 0 / 0 |
| **2016** | 0 / 0 / 0 / 0 / 0 | 0 / 0 / 0 / 0 / 0 | 0 / 0 / 0 / 0 / 0 | 0 / 0 / 0 / 0 / 0 |
